# Everyday Remembering During a Global Pandemic in Caring Dyads

**DOI:** 10.1093/geroni/igaa057.3457

**Published:** 2020-12-16

**Authors:** Emily Lustig, Alysha Naran, Ann Pearman, Christopher Hertzog

**Affiliations:** 1 Georgia Institute of Technology, Atlanta, Georgia, United States; 2 Georgia Institute of Technology, Atlanta, United States

## Abstract

During the COVID-19 global pandemic people’s lived experiences and day-to-day lives have been tremendously impacted. This impact is believed to be more severe in people with a memory-impaired partner at home. As part of an ongoing cognitive intervention project with the Emory-Georgia Tech Cognitive Empowerment Program (CEP), we conducted interviews with dyads (one person with diagnosed amnestic mild cognitive impairment (aMCI) and one person, in this case, a spouse who is an identified care partner). To address the COVID-19 pandemic, we supplemented the existing interview about everyday cognition with several questions about the dyadic experience during the pandemic. To date, we have conducted 5 qualitative interviews with dyads. Preliminary results indicate that the COVID-19 pandemic has created additional everyday challenges and cognitive burden for care partners of people diagnosed with aMCI. Some of these challenges include the need to manage pandemic precautionary behaviors, such as mask wearing and maintaining social distancing, for both themselves and the care recipient. In contrast, some aspects of everyday remembering among these dyads have improved (e.g. more advance planning of things like grocery shopping and outings). The results of these interviews will provide additional unique insights into the everyday cognitive challenges of the pandemic on caregivers and persons with aMCI.

